# A multicentre retrospective cohort study of ovarian germ cell tumours: Evidence for chemotherapy de-escalation and alignment of paediatric and adult practice

**DOI:** 10.1016/j.ejca.2019.03.001

**Published:** 2019-05

**Authors:** C. Newton, K. Murali, A. Ahmad, H. Hockings, R. Graham, V. Liberale, S.-J. Sarker, J. Ledermann, D.M. Berney, J. Shamash, S. Banerjee, S. Stoneham, M. Lockley

**Affiliations:** a Barts Health NHS Trust, West Smithfield, London, EC1A 7BE, UK; b University College Hospital, 235 Euston Road London, NW1 2BU, UK; c University Hospitals Bristol NHS Foundation Trust, Upper Maudlin Street, Bristol, BS2 8HW, UK; d The Royal Marsden Hospital, 203 Fulham Rd, Chelsea, London SW3 6JJ, UK; e The Wolfson Institute, CRUK Barts Cancer Centre, Queen Mary University London, Charterhouse Square, London EC1M 6BQ, UK; f Cancer Intelligence, Cancer Research UK, Angel Building, 407 St John Street, London EC1V 4AD, UK; g Centre for Molecular Oncology, Barts Cancer Institute, Queen Mary University of London, Charterhouse Square, London EC1M 6BQ, UK; h Research Department of Medical Education, UCL Medical School, Royal Free Campus, Hampstead, London NW3 2PR, UK; i University of Bristol, Senate House, Tyndall Avenue, Bristol BS8 1TH, UK

**Keywords:** Ovarian germ cell cancer, Dysgerminoma, Yolk sac tumour, Immature teratoma, Mixed germ cell tumour, BEP

## Abstract

**Background:**

Adult guidelines recommend BEP (bleomycin, etoposide, cisplatin) for all ovarian germ cell tumours, causing debilitating toxicities in young patients who will survive long term. Paediatricians successfully reduce toxicities by using lower bleomycin doses and substituting carboplatin for cisplatin, while testicular and paediatric immature teratomas (ITs) are safely managed with surgery alone.

**Aim:**

The aim was to determine whether reduced-toxicity treatment could rationally be extended to patients older than 18 years.

**Methods:**

Multicentre cohort study was carried out in four large UK cancer centres over 12 years.

**Results:**

One hundred thirty-eight patients were enrolled. Overall survival was 93%, and event-free survival (EFS) was 72%. Neoadjuvant/adjuvant chemotherapy (82% BEP) caused 27 potentially chronic toxicities, and one patient subsequently died from acute lymphoblastic leukaemia. There was no difference in histology, stage or grade in patients ≤/>18 years, and EFS was not different in these age groups (≤18:28% and >18:28%; log-rank *P* = 0.96). Histological subtype powerfully predicted EFS (log-rank *P* = 4.9 × 10^−7^). Neoadjuvant/adjuvant chemotherapy reduced future relapse/progression in dysgerminoma (n = 37, chemo:0% vs. no chemo:20%), yolk sac tumour (n = 23, 26.3% vs.75%) and mixed germ cell tumour (n = 32, 40%vs.70%) but not in IT (n = 42, 33% vs.15%). Additionally, we observed no radiological responses to chemotherapy in ITs, pathological IT grade did not predict EFS (univariate hazard ratio 0.82, 95% confidence interval: 0.57–1.19, *P* = 0.94) and there were no deaths in this subtype.

**Conclusion:**

Survival was excellent but chemotherapy toxicities were severe, implying significant overtreatment. Our data support the extension of reduced-toxicity, paediatric regimens to adults. Our practice-changing findings that IT was chemotherapy resistant and pathological grade uninformative strongly endorse exclusive surgical management of ovarian ITs at all ages.

## Highlights

• BEP (bleomycin, etoposide, cisplatin) chemotherapy caused multiple, severe and even life-threatening toxicities.
• Ovarian germ cell tumour subtypes were distinct diseases, requiring different management.
• Immature teratomas (ITs) were chemotherapy resistant.
• The World Health Organisation pathological grade of ITs did not predict outcome.
• There was no clinical difference in patients aged ≤/>18 years, but survival was worse in women >40 years (*P* < 0.02).

## 1 Introduction

Ovarian germ cell tumours (OvGCTs) are very rare, comprising only 1–2% of ovarian malignancies [1], [2]. They usually present at an early stage in children and young adults, are generally chemotherapy sensitive and outcomes are excellent even in metastatic disease [3], [4]. Fertility-sparing surgery is established as standard of care [5], [6], [7], but clinical trial data are severely lacking regarding the adjuvant management of these rare tumours in adult women, and progress has lagged behind other cancers [8].

OvGCTs are divided into histological subtypes according to the World health Organisation pathological classification: primitive germ cell tumours (dysgerminoma [Dys], yolk sac tumour [YST] and mixed germ cell tumour [MGCT]) and the teratomas (immature teratoma [IT] and mature teratoma [MT]) [9]. IT is further divided into three grades based on the amount of primitive neuroectodermal tumour (PNET). Prognosis varies by subtype, Dyss and teratomas having particularly favourable outcomes [10], [11]. Despite these differences, the National Comprehensive Cancer Network [6] recommends adjuvant BEP chemotherapy (bleomycin, etoposide and cisplatin) for all histologies in adults, except stage I Dys and stage I, grade 1 ITs. Recent European Society of Medical Oncology (ESMO) guidelines [7] describe active surveillance protocols for subsets of patients with stage I disease and normal postoperative tumour markers. BEP toxicities are significant, including pulmonary fibrosis, hypertension, permanent hearing damage, chronic kidney disease, Raynaud's phenomenon and an increase in secondary cancers such as leukaemia [12]. Late effects are particularly concerning in these young women with a very high likelihood of cure and some advocate close surveillance for all stage I OvGCTs, regardless of histology, with chemotherapy salvage if progression occurs [13], [14].

Reduced-toxicity treatments are already established in paediatric and male GCTs. JEB (carboplatin, etoposide and bleomycin) has replaced BEP in children [15], while adjuvant treatment of male seminomas (Dys equivalent) is with either carboplatin (AUC 7) or radiotherapy (20 Gy/10 fractions) [16], [17]. These strategies have not been tested in women. The most obvious discrepancy is in the management of ovarian ITs: adjuvant BEP is recommended for adults (except grade 1, stage IA) [6], whereas in children, evidence supports postoperative surveillance for all IT stages and grades [18]. This clearly presents a dilemma in the treatment of 18-year-old females [19]. The Malignant Germ Cell International Collaborative (MaGIC) conducted a retrospective analysis of IT data from paediatric (Children's Oncology Group and Children's Cancer and Leukaemia Group) and adult (Gynecologic Oncology Group) clinical trials [20]. Adults had higher stage, higher grade and lower complete debulking rates, and their overall survival (OS) was marginally lower (93% vs 99%), despite much higher administration of adjuvant chemotherapy (81 of 81 adult patients vs 8 of 98 children). Although it was not possible to determine whether the survival difference was due to trial eligibility or to disease biology, this analysis does question the overall efficacy of chemotherapy in ovarian ITs.

In view of the marked differences in adult and paediatric practice, the serious toxicities associated with current chemotherapy regimens and the paucity of available clinical data, we conducted a multicentre retrospective cohort study of adult and paediatric patients treated in four large UK cancer centres over 12 years. We interrogated the efficacy and toxicity of chemotherapy in the initial management of OvGCTs presenting in patients of all ages.

## 2 Methods

### 2.1 Patient selection

We analysed all OvGCTs at Barts Health NHS Trust, University College Hospital and University Hospitals Bristol NHS Foundation Trust, as well as adult patients presenting to the Royal Marsden Hospital, between 01 January 2005 and 31 December 2016. Hospital ethical approval was obtained, and data sharing agreements were established between partner organisations in accordance with the European General Data Protection Regulations. All primary gynaecological Dys, YST, IT and MGCT were identified from pathology and surgical databases. We excluded only one patient because of insufficient clinical information. Patient details were obtained via extensive review of hospital notes and computer records. Histology was reviewed centrally by supraregional specialists in each of the four individual cancer centres. All pathology reports were then reviewed again by an expert germ cell tumour pathologist (D.M.B.) to ensure accurate interpretation of local specialist pathology reports.

### 2.2 Statistical analysis

Statistical methods were documented in a prespecified statistical analysis plan. Univariable and multivariable Cox regression models were used to analyse the effects of covariates (age, Fédération Internationale de Gynécologie et d’Obstétrique (FIGO) stage, histology, grade, and chemotherapy) on the primary end-point, event-free survival (EFS). In the multivariable analysis, grade was excluded as it was not statistically significant and chemotherapy was also excluded as it was a non-randomised intervention rather than a baseline tumour characteristic. EFS was defined as the time from primary surgery until the date of documented relapse or progression of any germ cell histology (including disease progression while awaiting adjuvant chemotherapy). The age cut-off of paediatric and adult practice was ≤18 and > 18 years. The accepted upper age limit for adolescent and young adult (AYA) practice is 39 years, and thus, we defined a second age cut-off at ≥40 years to distinguish older adults from AYA. In view of the small patient numbers, neoadjuvant and adjuvant chemotherapies were combined for the purposes of statistical analysis and are collectively referred to here as ‘first-line chemotherapy’. Patients were censored on the date of the last follow-up or death. Schoenfeld residuals were examined to determine if Cox modelling was an appropriate analytical approach. Spearman's rank correlation was estimated pairwise for all variables. Kaplan–Meier survival curves were plotted, and *P*-values from the log-rank or Mantel–Haenszel test were reported. All applied statistical tests were two sided, and *P* < 0.05 was considered significant. Analyses were performed in R version 3.4.3 [21].

## 3 Results

### 3.1 Patient characteristics

We enrolled 138 patients (Table 1). One patient had primary endometrial MGCT, but the remainder had primary OvGCTs. Two patients with raised αFP (1178kIU/L and 42,000kIU/L) received neoadjuvant chemotherapy without diagnostic biopsy. Both were found to have MT at surgery after completion of chemotherapy and were grouped with MGCT for subsequent statistical analysis. There was a preponderance of early-stage disease, with only 38 stage III/IV patients (28% total). All but one patient had surgery, which was fertility sparing in 88% cases (120 of 137). The small number of patients who had bilateral salpingo-oophorectomy or hysterectomy were either older (≥40 years: 6 of 17 patients) or had undergone prophylactic surgery for underlying genetic syndromes (5 of 17 patients). A further six patients all had disease that had spread beyond the ovaries (FIGO II/III/IV).

**Table 1:** Baseline patient characteristics.

**Histology**	**Number**
Dysgerminoma	37
Yolk sac tumour	23
Immature teratoma	42
PNET	4
Mixed germ cell tumour	32
Total	138
**Age of patients**	**Median (range)**
Dysgerminoma	21 (11–47)
Yolk sac tumour	27 (14–69)
Immature teratoma	26 (11–44)
PNET	23 (9–32)
Mixed germ cell tumour	24.5 (8–76)
All patients	23.5 (8–76)
**First-line chemotherapy**	**n (%)**
Dysgerminoma	22 (59%)
Yolk sac tumour	19 (82%)
Immature teratoma	9 (21%)
PNET	4 (100%)
Mixed germ cell tumour	22 (69%)
All patients	76 (55%)
**FIGO stage**	**Number**
IA/B	59
IC	27
II	11
III	23
IV	15
Not known	3
Total	138
**First-line chemotherapy**	**n (%)**
IA/B	10 (17%)
IC	18 (66%)
II	9 (82%)
III	23 (100%)
IV	14 (93%)
Not known	2 (67%)
Total	76

### 3.2 Overall and event-free survival

**Fig. 1:**
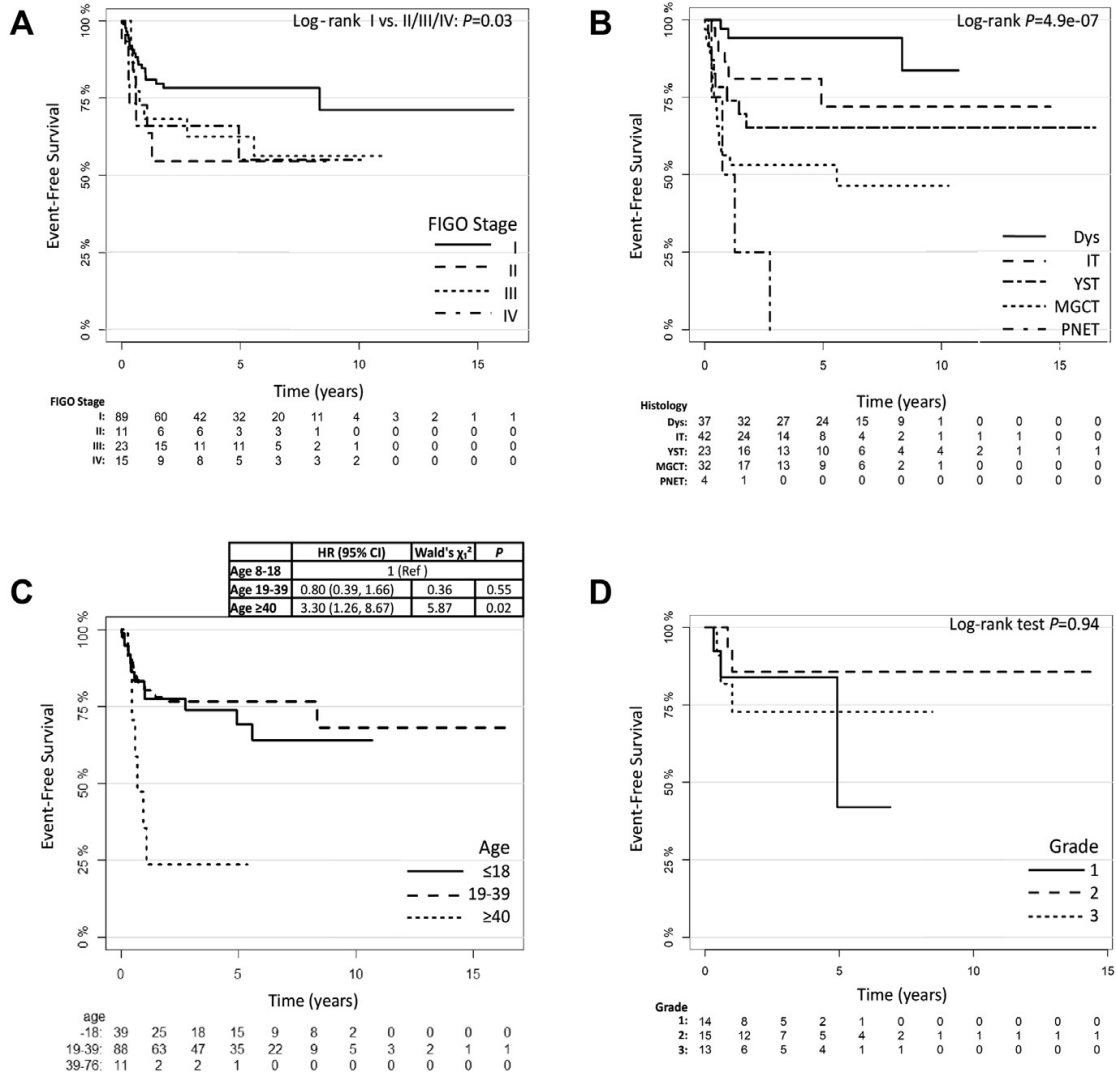
Kaplan–Meier event-free survival. Event-free survival according to (A) FIGO stage, (B) histological OvGCT subtype, (C) patient age at diagnosis and (D) grade of immature teratoma. OvGCT, ovarian germ cell tumour; Dys, dysgerminoma; IT, teratoma; YST, yolk sac tumour; MGCT, mixed germ cell tumour; PNET, primitive neuroectodermal tumour; HR, hazard ratio; CI, confidence interval.

Median follow-up was 56.6 months (range: 11 days-16.5 years). The OS rate was 93%. Nine patients (7%) died of disease and one died of acute lymphoblastic leukaemia (ALL), which was presumed to be treatment related. EFS was 72%, and relapse/progression events occurred in 39 patients (28%). As expected, residual disease after surgery was associated with disease relapse (Suppl. Fig. 3; log-rank test *P* = 3.0 × 10^−5^). In the Cox regression analysis (Table 2), neither age (univariable hazard ratio [HR] 1.03, 95% confidence interval [CI] 1.0–1.05, *P* = 0.09; multivariable *P* = 0.25) nor FIGO stage (univariable HR 1.31, 95% CI 1.00–1.704, *P* = 0.051; multivariable *P* = 0.53) was significantly associated with EFS, although EFS was significantly different when FIGO stage I was compared with all other stages combined (Fig. 1A; log-rank test *P* = 0.03). In contrast, histological subtype strongly predicted outcome (Table 2; univariable *P* = 5.8 × 10^−5^ and Fig. 1B; log-rank test *P* = 4.9e-07).

**Table 2:** Univariable and multivariable Cox regression analysis.

Predictor	Univariable				Multivariable		
	HR (95% CI)	LR χ^2^ test	d.f., *p*-value	c-index	HR (95% CI)	Δχ^2^ (d.f.)	*p*-value
Histology, Dys	1 Ref.	24.68	4, 5.8e-05	0.73	1 Ref.	24.69 (4)	5.8e-05
Histology, IT	3.02 (0.80, 11.46)				3.15 (0.82, 12.06)		
Histology, YST	5.06 (1.34, 19.09)				5.56 (1.41, 21.96)		
Histology, MGCT	8.87 (2.57, 30.55)				10.73 (2.79, 41.21)		
Histology, PNET	19.69 (4.35, 89.10)				26.093 (5.27, 129.18)		
Age (years)	1.03 (1.0, 1.05)	2.84	1, 0.09	0.53	0.934 (0.87, 1.00)	1.32 (1)	0.25
Chemotherapy, yes	0.84 (0.45, 1.58)	0.29	1, 0.59	0.53			
Stage (linear)	1.31 (1.01, 1.70)	3.82	1, 0.05	0.59	0.45 (0.22, 0.94)	0.40 (1)	0.53
Grade	0.824 (0.570, 1.193)	1.17	1, 0.28	0.55			
Age*stage					1.03 (1.01, 1.05)	6.86 (1)	0.01
LR χ^2^ test (d.f., *p*)					33.265 (7, 2.4e-05)		
c-index (95% CI)					0.770 (0.677, 0.864)		

HR, hazards ratio, CI, confidence interval, LR χ^2^, log-rank chi-squared test, d.f., degrees of freedom, c-index, Harrell's c-index; Dys, dysgerminoma; IT, teratoma; YST, yolk sac tumour; MGCT, mixed germ cell tumour; PNET, primitive neuroectodermal tumour.

### 3.3 Age

Adult patients (>18 years) made up 72% of cases (99 of 138). A greater proportion of women older than 18 years received chemotherapy (46% ≤ 18 vs. 59% > 18), despite there being no difference in the disease stage (Table 3; proportions test χ^2^ = 5.87, d.f. = 3, *P* = 0.12) or histological subtype (Table 3; proportions test χ^2^ = 4.06, d.f. = 4, *P* = 0.40) when patients ≤18 and > 18 years were compared. There was no significant difference in EFS between these age groups (log-rank *P* = 0.96, not shown).

**Table 3:** Patient characteristics by predefined age cut-off.

	Patient's age		
	≤18 years, *N* (%)	>18 years, *N* (%)	≥40 years, *N* (%)
Total patient number (138)	39 patients	99 patients	11 patients
Stage			
I	25 (64%)	61 (62%)	3 (27%)
II, III, IV	14 (36%)	35 (35%)	7 (64%)
NK	0	3 (3%)	1 (9%)
Proportions test	χ^2^ = 4.06, d.f. = 3, *p*-value = 0.12		
Histology			
Dys	12 (31%)	25 (25%)	1 (9%)
IT (total)	15 (38%)	27 (27%)	1 (9%)
Gd 1	7	7	1
Gd 2	2	13	0
Gd 3	6	7	0
YST	6 (15%)	17 (17%)	2 (18%)
PNET	1	3	0
MT	1	1	0
MGCT	4 (10%)	26 (26%)	7 (64%)
Proportions test	χ^2^ = 4.06, d.f. = 4, *p*-value = 0.40		

χ^2^ ,chi-squared test, d.f., degrees of freedom; Dys, dysgerminoma; IT, teratoma; YST, yolk sac tumour; MGCT, mixed germ cell tumour; PNET, primitive neuroectodermal tumour; MT, mature teratoma.

Only 11 women were ≥40 years old. Most women ≥40 years presented with higher stage disease (Table 3), and they were more likely to receive first-line chemotherapy (73% ≥ 40 compared with 55% overall). Despite this, their EFS was significantly worse than younger patients (Fig. 1C; HR 3.30, 95% CI 1.26–8.67, *P* = 0.02) and OS rate was only 55% (5 of 11 women ≥40 years died of their disease) compared with 93% for the entire cohort.

### 3.4 Chemotherapy use and toxicity

First-line chemotherapy (neoadjuvant or adjuvant) was administered to 55% patients (76 of 138) and was more likely to be given in higher stage disease (Table 1). Chemotherapy was most commonly adjuvant (79%). Only 16 patients received neoadjuvant chemotherapy, 12 of whom had stage III/IV disease (Suppl. Fig. 1). BEP was used most frequently, accounting for 82% (62 of 76) of cases, 11% received JEB (8 of 76, all aged ≤18 years) and only one patient with IIIC PNET received a non-platinum–containing regimen (VIDE: vincristine, ifosfamide, doxorubicin and etoposide).

Chemotherapy caused significant short- and long-term toxicity (Table 4). This retrospective review will have only identified severe, clinically significant toxicities and so likely underestimates the true extent of treatment-related side-effects. Nonetheless, we observed 27 potentially chronic toxicities in 22 patients including kidney injury (n = 2), cardiac failure (n = 1), tinnitus or hearing loss (n = 9), bleomycin lung toxicity (n = 4) and peripheral neuropathy (n = 11). One 14-year-old patient died of ALL 2 years after surgery and adjuvant JEB for stage IIIA MGCT (Dys and YST). In view of these severe toxicities, and the powerful influence of histology on EFS (Table 2 and Fig. 1B), we investigated the use and efficacy of chemotherapy according to histological subtype.

**Table 4:** The number of occurrences of potentially chronic chemotherapy treatment–related toxicities.

Potentially chronic toxicity	Number
Kidney injury	2
Cardiac failure	1
Tinnitus/hearing loss	9
Bleomycin lung	4
Peripheral neuropathy	11
died of ALL	1
Total	27

ALL, acute lymphoblastic leukaemia.

### 3.5 Immature teratoma

IT was the most common histology (Table 1; 42 cases), and this subtype was overwhelmingly low stage (32 of 42 patients stage IA/B) (Suppl. Fig. 2A). Most patients were safely managed with surgery alone, and there were no deaths in this group. Only nine of 42 patients with ITs received first-line chemotherapy (Table 1), three preoperatively (stage III, IV and unknown; discussed in the following section) and six postoperatively (four stage IA and two stage IC) treated patients. The incidence of any teratoma recurrence (IT, MT and gliomatosis peritonei) was not reduced by first-line chemotherapy, occurring in three of nine (33%) chemotherapy-treated patients compared with five of 33 (15%) chemotherapy-naïve patients (Suppl. Tables 1 and 2). Because this could be explained by the higher disease stage of chemotherapy-treated patients (only three patients with ITs did not have stage I disease and all three received chemotherapy, Suppl. Fig. 2A), we examined chemotherapy efficacy in the four patients with ITs with measurable disease prior to chemotherapy. This included the three neoadjuvant patients listed previously and one chemotherapy-naïve patient who relapsed after initial surgery.

A 34-year-old woman relapsed in the brain and two other distant sites one year after initial surgery for stage IA, grade 2 ITs. The brain mass was resected (grade 2 ITs) with residual disease at the operation site. She received EP-OMB chemotherapy (etoposide, cisplatin, vincristine, methotrexate and bleomycin) and intrathecal methotrexate with minor response on MRI brain but no radiological response in the other two metastatic sites. Both these metastases were resected (again grade 2 ITs), and she received postoperative ACE chemotherapy (actinomycin, cyclophosphamide, etoposide). Her most recent MRI brain was stable, and she was well at censor, four years after her most recent surgery.

The other three patients (stage III, IV and unknown) were all treated preoperatively. Two had no radiological response to chemotherapy, and IT was confirmed on subsequent surgical debulking. The only evidence of any chemotherapy response in ITs was in a 37-year-old individual presenting with abdominal pain, a rapidly growing pelvic mass and urinary sepsis. Presentation lactate dehydrogenase was 916, and a scanty diagnostic biopsy of the mass revealed PNET elements consistent with grade 3 ITs and a possible accompanying Dys, suggesting that this patient might have actually had a MGCT. Three cycles of BEP achieved only a 25% radiological reduction and surgical debulking revealed MT.

### 3.6 IT grade

Relapse and progression with any teratoma histology (immature, mature and gliomatosis) occurred in all grades of ITs (Gd1: 3/14, Gd2: 2/15 and Gd3: 3/13). Additionally, immature elements were found at recurrence in patients who initially presented with all three grades of ITs (Gd1: 1/14, Gd2:1/15 and Gd3: 1/13). Strikingly, pathological IT grade did not predict EFS (Table 2; univariable HR 0.82, 95% CI 0.57–1.19, *P* = 0.28: Fig. 1D; log-rank *P* = 0.94).

### 3.7 Primitive germ cell tumours (Dys, YST and MGCT)

In marked contrast to the chemotherapy resistance of ITs, radiological responses were observed in 10 of 10 pure Dys and four of five pure YST chemotherapy-naïve patients. Dys outlook was exceptionally good, with no patient deaths and no relapses in any patient after first-line chemotherapy (Suppl. Fig. 2B; 22 of 22 patients). Relapse did occur in three of 15 patients with stage IA/B Dys who were initially treated with surgery alone. All three were salvaged with platinum-containing chemotherapy (Suppl. Table 1), and all were alive and disease free 16, 65 and 117 months after relapse. Only four patients with YST did not receive first-line chemotherapy: two stage IA and two stage IC (Suppl. Fig. 2C). Of these four, three progressed very rapidly after initial surgery at 53, 68 and 103 days. As with Dys, chemotherapy response in YST was excellent with all three of these chemotherapy-naïve relapses being successfully salvaged with BEP. All three patients were disease free 31, 28 and 26 months after diagnosis of recurrence.

Further evidence of chemotherapy sensitivity in YST came from the observation that relapse/progression appeared to be reduced by chemotherapy overall (5 of 19; 26% chemo-treated vs. 3 of 4; 75% chemo-naïve; Suppl. Table 1) and that median time to relapse was increased by first-line chemotherapy treatment (344 days in chemotherapy-treated patients compared with 68 days in chemotherapy-naive patients; unpaired *t*-test, *P* = 0.07). Re-treatment of the five patients with YST who relapsed after BEP treatment was less successful. Only two of these five patients were salvaged by further surgery and chemotherapy but both were still in remission at the censor date, two and eight years after diagnosis of relapse. The remaining three relapsed patients eventually died of their disease. Two of these three deaths occurred in the only two patients with YST who were aged ≥40 years.

All but one MGCT had a yolk sac component to their histology (29 of 30). Most patients (20 of 30) received first-line chemotherapy (Suppl. Fig. 2D). Eight of these chemotherapy-treated patients subsequently relapsed (Suppl. Table 1), but the pathology of six of these relapses revealed MTs and mature glial tissue only and these patients were all salvaged with surgery alone. Four MGCT patients died of disease. One 19-year-old patient with stage IV disease died of rapid disease progression postoperatively before initiation of adjuvant chemotherapy. The other three deaths were all in women aged ≥40 years. Two of these patients relapsed with confirmed YST (αFP rise ± biopsy) and were not salvaged by second-line treatment, while the third declined further chemotherapy.

### 3.8 Primitive neuroectodermal tumours

Somatic transformation of ovarian teratomas is associated with a poor outcome. Five patients had PNET, four at diagnosis and one at relapse. Only one was successfully treated to complete remission with surgical resection followed by GAMEC chemotherapy (GCSF, actinomycin, methotrexate, etoposide, cisplatin). She was disease free 5 years after diagnosis, confirming that PNET can be salvaged with intensive combination treatment [22].

## 4 Discussion

OvGCTs are a heterogenous group of rare diseases. Clinical data are severely lacking, and progress has lagged behind other cancers [8]. Paediatricians aim to reduce late effects in these patients with a near certainty of long-term cure, but until now, treatment-induced toxicities have not been prioritised in adult practice, and international guidelines still recommend BEP in nearly all cases.

This cohort study is one of the few large studies to investigate these exceptionally rare ovarian cancers. The OS rate was excellent at 93% with a median follow-up of 56.6 months. However, we recorded 27 potentially chronic toxicities in 76 chemotherapy-treated patients and one probable chemotherapy-related death. Together, this implies widespread overtreatment, and so, reduction of BEP use is a priority. Recent ESMO guidelines [7] now support surveillance of subsets of patients with stage I OvGCTs. Four of the 22 patients who experienced potentially chronic toxicities had stage I disease and thus might have been spared chemotherapy altogether according to these new guidelines. This approach appeared safe in our series. We identified 25 patients with stage I Dys, YST and MGCT who were initially managed without chemotherapy. Although 10 of these subsequently relapsed, all were salvaged with platinum-containing chemotherapy (BEP/JEB in nine patients) with or without further surgery. In agreement with others [23], our study therefore endorses and extends this expectant policy by advocating the avoidance of chemotherapy in all FIGO stage I patients.

In higher stage disease, paediatricians have already reduced chemotherapy toxicity by the widespread and accepted use of JEB in preference to BEP. We identified no statistically significant differences in OvGCTs presenting in patients younger and older than 18 years. Comparable series have made similar observations [24], [25], providing a compelling rationale for also using carboplatin-based chemotherapy in adult patients. This approach has already been shown to be equivalent in Dys [26] and is currently being tested in other patients aged 11–25 years with other ovarian germ cell histologies in the AGCT1531 study [27]. In contrast, women older than 40 years appeared to present with higher stage disease and were more likely to receive first-line chemotherapy, and their EFS and OS were significantly worse than younger adults. Age over 40 years has previously been identified as a poor prognostic factor [28], and clinical trials should now determine whether this reflects suboptimal treatment or underlying disease biology with the ultimate aim of improving the survival of this group of women.

Our most important and practice-changing finding was that, compared to the excellent responses to chemotherapy observed in the primitive germ cell tumours, we found little evidence of response to chemotherapy in ITs. Although IT relapse and progression occurred frequently, there were no deaths either in pure ITs or in teratomatous relapse of MGCT and both were effectively managed with surgery alone. Eight of the nine patients with ITs who received chemotherapy were >18 years. We now propose that these adult patients might be spared the risks of BEP chemotherapy and managed safely with surgery as is currently the case in paediatric practice and for MT at all ages.

This clinical overlap between IT and MT is supported by frequent genetic homozygosity in IT and MT, but not in MGCT, indicating a common cellular origin of IT and MT with likely biological similarities in these histologies [29]. Developmental differences between ITs and other OvGCT subtypes can also be inferred from their more stable copy number profile and absence of 12p gain [30]. Furthermore, the observation that copy number profiles do not differ according to the grade of ITs [30] provides a biological explanation for our intriguing observation that EFS was identical regardless of the IT grade. This provocative finding differs from a previous pooled analysis of clinical trial data by MaGIC in which IT grade did predict future relapse [20]. This discrepancy could be explained by small numbers and retrospective analysis in both studies, and clinical trials are required to resolve this issue. Nonetheless, we agree with MaGIC that there was little evidence of chemotherapy response in any grade of ITs and thus question the utility of the World Health Organisation pathological IT grade as a biomarker to direct patient care.

Finally, we confirmed that Dys was exquisitely chemotherapy sensitive with clear evidence of radiological response to chemotherapy, no relapses after chemotherapy and no deaths at a median follow-up of 6.38 years. Later relapse of this histology is possible with a high expectation of chemotherapy salvage as was the case for one patient in our cohort. Single-agent, high-dose carboplatin may therefore be an appropriate and less toxic treatment for Dys, as is already established in the histologically equivalent testicular seminoma.

In summary, our large, multicentre retrospective cohort study has exposed significant overtreatment with excellent survival but frequent debilitating and potentially life-threatening toxicities. We have shown that platinum-containing chemotherapy can safely be avoided in stage I disease and that the role of chemotherapy in the management of ovarian ITs should be reviewed. Treatment-induced toxicities would be further reduced by using single-agent carboplatin in Dys, and based on the clinical similarity between patients older and younger than 18 years, we propose that clinical trials should now compare BEP with carboplatin-based chemotherapy, such as JEB, in YST-containing histologies at all ages. These practice-changing recommendations will herald a new era in the management of these inherently good prognosis cancers.

## Conflicts of interest statement

None declared.
