# Supplementary material for: A multicentre retrospective cohort study of ovarian germ cell tumours: Evidence for chemotherapy de-escalation and alignment of paediatric and adult practice
Source: Eur J Cancer. 2019 May;113:19–27. doi: 10.1016/j.ejca.2019.03.001 (PMC6522056; doi:10.1016/j.ejca.2019.03.001)

Suppl. Fig 3. Kaplan-Meier Event-Free Survival (EFS) according to residual disease

No = no macroscopically visible residual disease, Yes = any macroscopically visible residual disease at completion of surgery

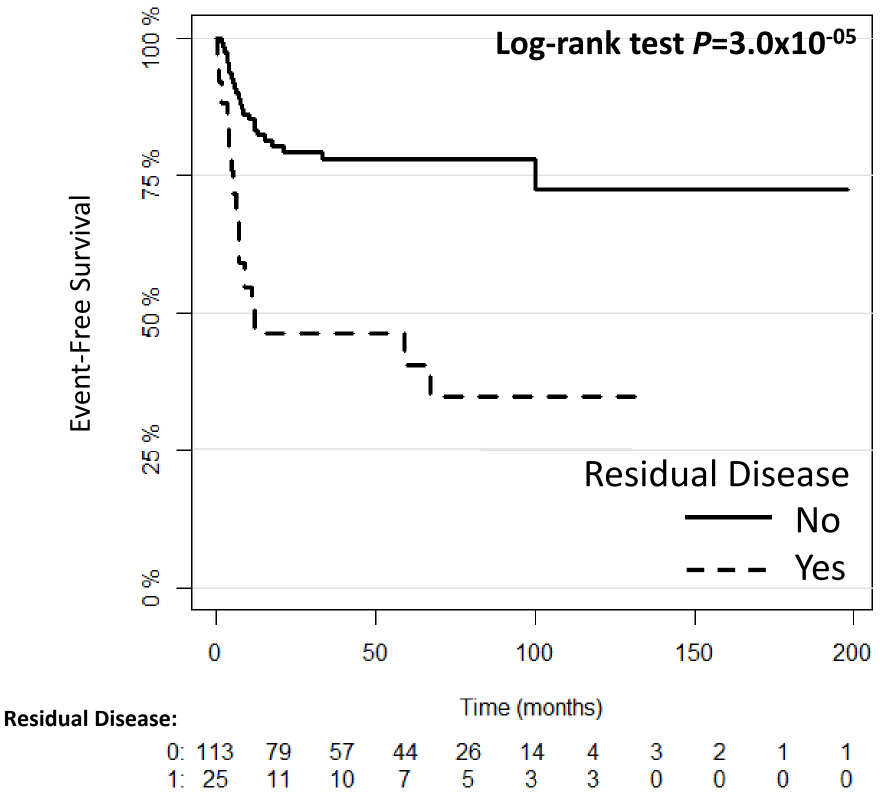

Supplement: Multimedia component 1 [file mmc1.pdf]
