# Supplementary material for: A multicentre retrospective cohort study of ovarian germ cell tumours: Evidence for chemotherapy de-escalation and alignment of paediatric and adult practice
Source: Eur J Cancer. 2019 May;113:19–27. doi: 10.1016/j.ejca.2019.03.001 (PMC6522056; doi:10.1016/j.ejca.2019.03.001)

Suppl. Fig 1. Number of patients receiving neo-adjuvant and adjuvant chemotherapy according to FIGO stage

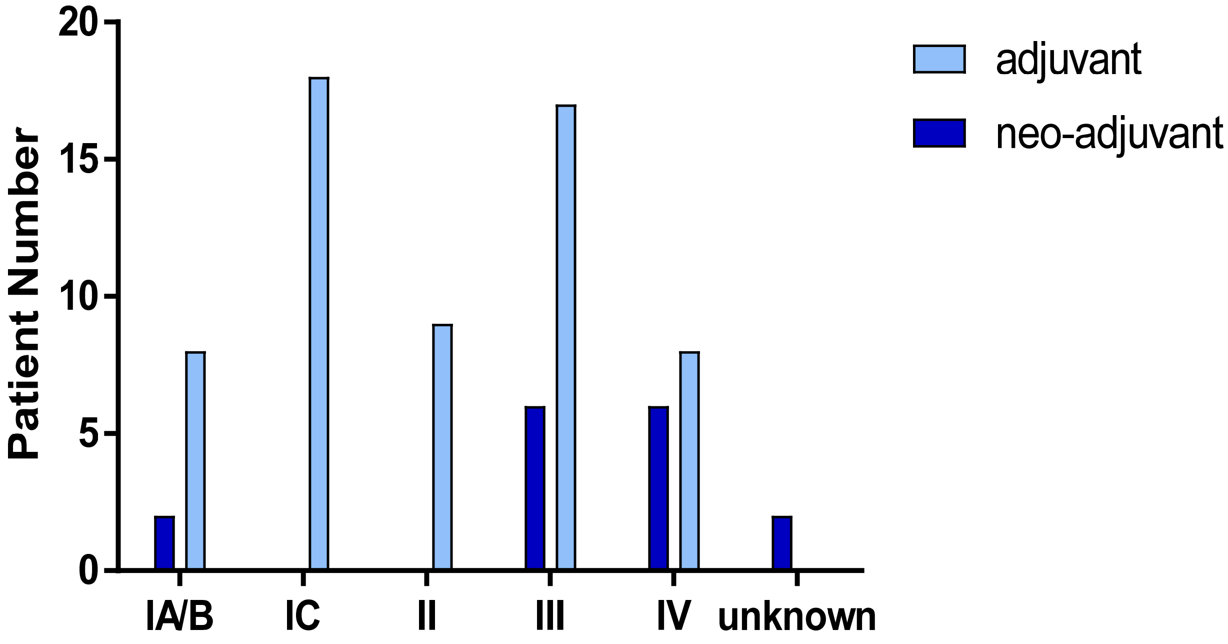

Supplement: Multimedia component 2 [file mmc2.pdf]
