# Supplementary material for: A multicentre retrospective cohort study of ovarian germ cell tumours: Evidence for chemotherapy de-escalation and alignment of paediatric and adult practice
Source: Eur J Cancer. 2019 May;113:19–27. doi: 10.1016/j.ejca.2019.03.001 (PMC6522056; doi:10.1016/j.ejca.2019.03.001)

Suppl. Fig 2. Number of patients receiving first-line chemotherapy by FIGO stage and histology

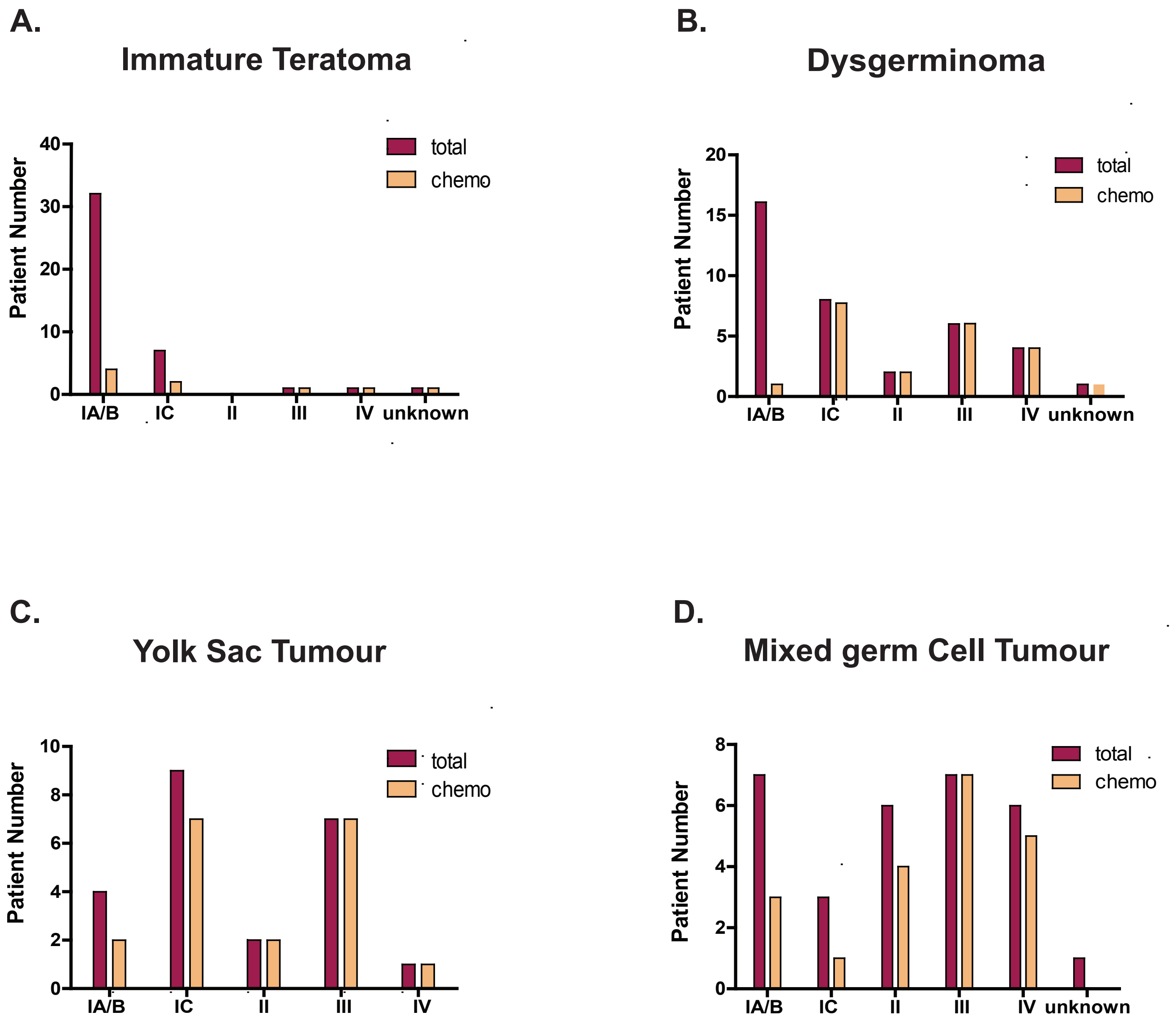

Supplement: Multimedia component 3 [file mmc3.pdf]
