# Supplementary material for: A multicentre retrospective cohort study of ovarian germ cell tumours: Evidence for chemotherapy de-escalation and alignment of paediatric and adult practice
Source: Eur J Cancer. 2019 May;113:19–27. doi: 10.1016/j.ejca.2019.03.001 (PMC6522056; doi:10.1016/j.ejca.2019.03.001)
Supplement: Multimedia component 4 [file mmc4.docx]

**Suppl. Table 1. Relapse/progression events following first-line chemotherapy**

| Chemotherapy - No | | | | |  | Chemotherapy - Yes | | | | |
| --- | --- | --- | --- | --- | --- | --- | --- | --- | --- | --- |
| Histology | Event: No | Event: Yes | Total | **% with event** |  | Histology | Event: No | Event: Yes | Total | **% with event** |
| Dys | 12 | 3 | 15 | **20%** |  | Dys | 22 | 0 | 22 | **0%** |
| IT | 28 | 5 | 33 | **15.10%** |  | IT | 6 | 3 | 9 | **33.33%** |
| YST | 1 | 3 | 4 | **75%** |  | YST | 14 | 5 | 19 | **26.30%** |
| MGCT | 3 | 7 | 10 | **70%** |  | MGCT | 12 | 8 | 20 | **40%** |
| PNET | 0 | 0 | 0 | **NA** |  | PNET | 0 | 4 | 4 | **100%** |
| Total | 44 | 18 | 62 | **29%** |  | Total | 55 | 21 | 76 | **27.60%** |
