# Supplementary figures and images for: A multicentre retrospective cohort study of ovarian germ cell tumours: Evidence for chemotherapy de-escalation and alignment of paediatric and adult practice
Source: Eur J Cancer. 2019 May;113:19–27. doi: 10.1016/j.ejca.2019.03.001 (PMC6522056; doi:10.1016/j.ejca.2019.03.001)

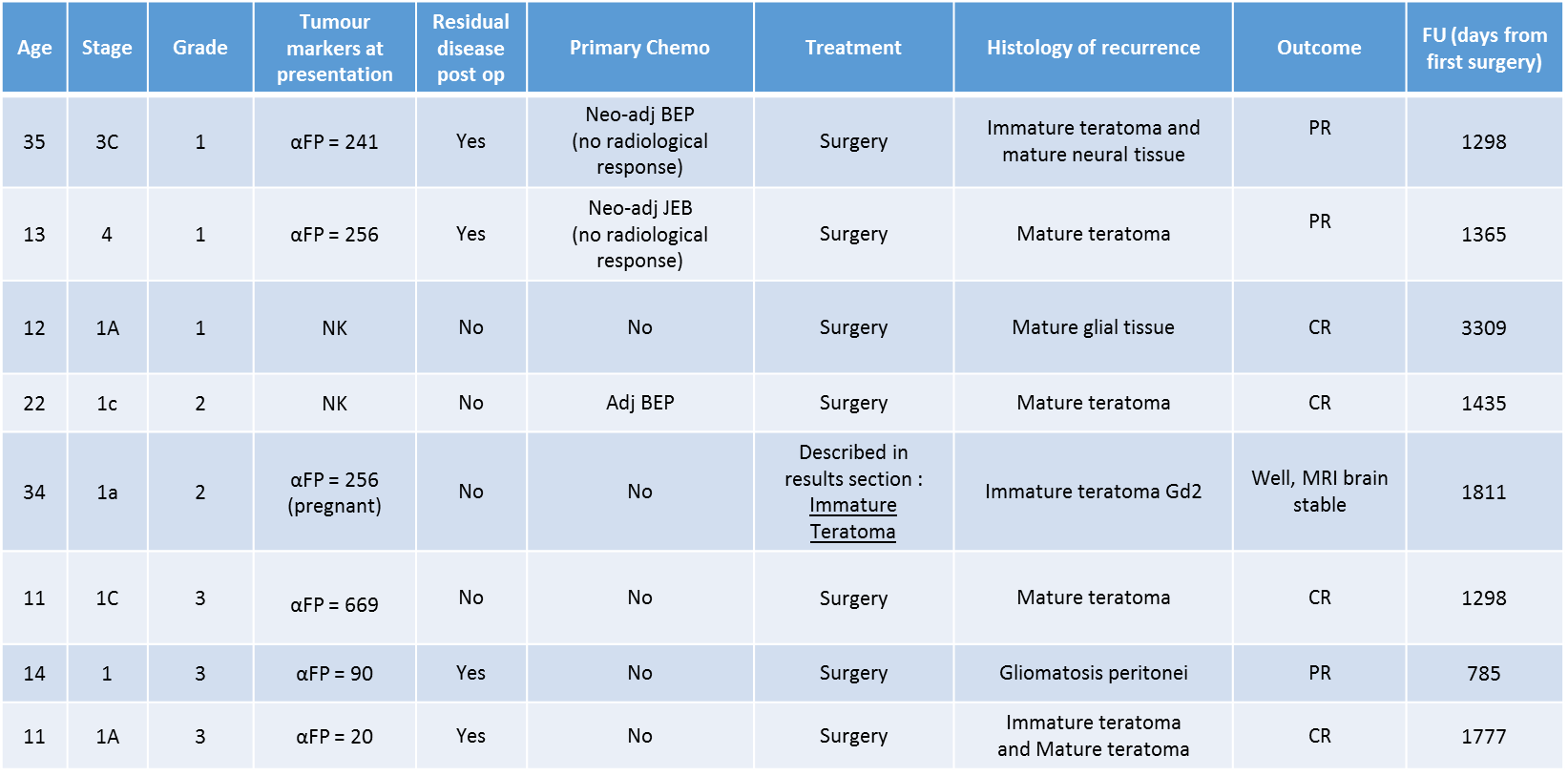


**Suppl. Table 2. Outcomes of patients with relapsed Immature Teratoma**

Supplement: Multimedia component 5 [file mmc5.docx]
